# U.S. trends of anti-vascular endothelial growth factor use from 2017–2023: An analysis of medicare, medicaid, and commercial insurance

**DOI:** 10.1371/journal.pone.0335390

**Published:** 2026-01-13

**Authors:** Kiran Malhotra, Joseph Colcombe, Sachi Patil, Daniel Vail, Ravi Parikh

**Affiliations:** 1 Department of Ophthalmology, NYU Grossman School of Medicine, New York, New York, United States of America; 2 Department of Health Informatics, NYU Langone Health, New York, New York, United States of America; 3 New York Eye and Ear Infirmary, New York, New York, United States of America; 4 Manhattan Retina and Eye Consultants, New York, New York, United States of America; Tsukazaki Hospital, JAPAN

## Abstract

**Purpose:**

Anti-vascular endothelial growth factor medications are a cornerstone in the treatment of many macular diseases in modern ophthalmology. These medications accrue significant economic burdens to individuals and health systems, and recent data on health-system level practice patterns involving agent selection is lacking. We sought to examine utilization of intravitreal anti–VEGF agents from 2017 to 2023 across various payors and diagnoses in the United States in order to analyze trends in usage.

**Methods:**

A retrospective cross-sectional study was performed*.* Data were obtained from the Epic Cosmos database, comprised of de-identified data from the electronic health record, Epic, of 238 million patients from all 50 states from January 1, 2017 to December 31, 2023. Encounters where a patient received an intravitreal injection in an ophthalmology clinic were categorized by intravitreal medication ordered, visit diagnosis, and primary insurance payor. The overall usage rates of each medication across different diagnoses and primary payor were compared. Total number of injections per year of anti-VEGF medication, the rate per 100000 ophthalmology encounters (OE), and the rate in different retinal conditions and with various insurance providers were the main outcome measures.

**Results:**

From 2017–2023, there were 571,545 anti-VEGF injections administered, with 53.6% aflibercept 2 mg (306,247/571,545; 625.8/000 OE), 34.8% bevacizumab 1.25 mg (198,696/571,545; 414.7/100000 OE), 7.83% ranibizumab 0.3 mg and 0.5 mg (44,803/571,545; 99.2/100000 OE), and 3.43% faricimab 6 mg (19,624/571,545; 32.8/100000 OE). Brolucizumab 6 mg, ranibizumab-eqrn 0.5 mg, ranibizumab-nuna 0.5 mg, and aflibercept 8 mg accounted for <1% of all injections. From 2017–2023, the rate of aflibercept 2 mg increased from 361.3 injections/100000 OE to 913.1, bevacizumab increased from 274.1 to 538.1, and ranibizumab decreased from 92.4 to 64.0. The rate of faricimab increased from 37.9/100000 OE to 189.9 from 2022–2023. Patients with Medicare received aflibercept 2 mg at higher rates than bevacizumab (364.3/100000 OE vs 187.1, respectively) [t(6) = 5.67, p = 0.001] and faricimab (19.4/100000 OE), a pattern not seen in commercially insured [t(6)=2.07, p > 0.05] patients. Medicaid patients had a marginally significant difference in use of bevacizumab (M = 19.9/100000 OE, SD = 7.32) and aflibercept 2 mg (16.1/100000 OE, SD = 7.22) [t(6)]=2.95, p = 0.026].

**Conclusions:**

Aflibercept 2 mg was the most common drug used overall from 2017–2023. Faricimab had the highest absolute and relative increase in utilization from 2022–2023. Patients with Medicare B or C were significantly more likely to receive aflibercept over bevacizumab.

## Introduction

Intravitreal injections of anti-vascular endothelial growth factor (anti-VEGF) agents are cornerstone therapies in ophthalmology and the mainstay of treatment for many retinal diseases, such as neovascular age-related macular degeneration (nAMD), diabetic retinopathy (DR), diabetic macular edema (DME), retinal vein occlusions (RVO), and other retinal neovascular diseases. Globally, anti-VEGF demand and utilization is rising, with a significant cost burden on health systems, especially in developing countries [1].

Currently, ranibizumab (Lucentis; Genentech, South San Francisco, CA), aflibercept 2 mg (Eylea; Regeneron, Tarrytown, NY), and relative newcomers aflibercept 8 mg (Eylea HD; Regeneron, Tarrytown, NY), brolucizumab (Beovu, Novartis, Basel, Switzerland), and faricimab (Vabysmo; Genentech/Roche, Basel, Switzerland), are in-market anti-VEGF medications with United States Food and Drug Administration (FDA) approved ophthalmologic indications [2,3]. Brolucizumab first received FDA approval in October 2019 for nAMD, though its adoption in America may be hampered by numerous published cases of retinal vasculitis and vascular occlusion associated with the medication [4]. Faricimab obtained FDA approval in January 2022 for nAMD and DME, and aflibercept 8 mg (Eylea HD) was approved in August 2023 for those same indications, along with DR. In the current marketplace, ranibizumab, aflibercept 2 mg, and faricimab are FDA-approved for diabetic macular edema and diabetic retinopathy, neovascular macular degeneration, and macular edema following retinal vein occlusion. Aflibercept 8 mg is approved for neovascular macular degeneration and diabetic macular edema and is pending approval for macular edema secondary to retinal vein occlusion. Brolucizumab is also FDA-approved for the above two indications (the clinical trials related to retinal vein occlusion were discontinued prematurely after reports of retinal vasculitis during the nAMD trials, as noted above). It should be noted that bevacizumab, despite its widespread use, is not FDA-approved for the treatment of any ophthalmologic disease, and all intravitreal use is considered off-label by the FDA.

Prior data examining national utilization across all diagnoses identified bevacizumab as the most used anti-VEGF agent for nAMD, DR, and RVO from 2006–2015 [5]. Additionally, ranibizumab use was found to be declining after 2014 across all indications, while aflibercept utilization (both absolute volume of injections and injection rate per patient) appeared to be rapidly increasing, with nAMD the most common indication for all anti-VEGF agents [5]. Bevacizumab, while still the leading anti-VEGF agent for nAMD patients in 2015, had declined from a peak in 2012 [5].

That analysis predated more recent critical studies involving anti-VEGF agents, such as Diabetic Retinopathy Clinical Research Network (DRCR) Protocol T, which demonstrated the superiority of aflibercept compared to bevacizumab and ranibizumab among eyes with DME and a baseline acuity worse than 20/40 [6], or Protocol S, or the CLARITY trial, which demonstrated the non-inferiority of anti-VEGF treatment (ranibizumab and aflibercept, respectively) compared to panretinal photocoagulation (PRP) in proliferative diabetic retinopathy [7,8]. Indeed, in the years following the publication of Protocol S, PRP rates decreased, while anti-VEGF rates increased for the treatment of PDR, implicating the influence of Protocol S and other randomized control trials in altering clinical practice patterns if practically feasible for clinicians and patients [9]. The effect of pivotal clinical trials involving brolucizumab (FDA approval first received in 2019), faricimab (2022), ranibizumab biosimilars (2021) and aflibercept 8 mg (2023), all approved after 2015, on anti-VEGF patterns has not yet been studied.

Important data from a more recent study, published in *Ophthalmology* in 2020, suggests aflibercept has slightly surpassed bevacizumab in total market share, and that ranibizumab has a relatively small market share [10]. However, no agent comprised over 50% market share in those data, as bevacizimab did from the combined years 2006–2015. Additionally, those data only included Medicare Part B claims from one-year (2018) [10]. Thus, data on faricimab, aflibercept 8 mg, brolucizumab, and ranibizumab biosimilars are still sparse.

The United States spends more on healthcare than other high-income economies, and a major contributor is the price of pharmaceuticals (including anti-VEGF agents) [11]. While the volume of anti-VEGF intravitreal injections is rising, ophthalmic medication prices in the United States have remained relatively stable and have not followed the same trends of declining prices in other high-income nations [6,12]. As the aforementioned newer anti-VEGF medications enter the treatment space, the influence of the cost burden of these medications (aflibercept 8 mg and 2 mg single use-vials have wholesale acquisition costs [WAC] of $2,625 and $1,957.5 respectively, a standard faricimab 6 mg single-use dose has a WAC of $2,190, and a standard brolucizumab 6 mg single-use dose has a WAC of $2,036) on implementation patterns remains to be seen [13–15]. These trends may become particularly important with the growth of the population over 65 and rising number of patients with diabetes [16,17].

Another change to the anti-VEGF market is the expiration of the U.S. patents for bevacizumab (June 2019), and ranibizumab (June 2020), and likely aflibercept (June 2023–2027, though under heavy litigation) [18]. Biosimilars of these medications are currently under active development and may soon enter the treatment sphere, adding another layer to market dynamics and consumer trends. Mixed predictions exist on the economic ramifications of these up-and-coming therapeutic options [19,20].

The COVID-19 pandemic also played a role in changing anti-VEGF practice patterns. The pandemic, especially during global lockdowns in the earlier phase from 2019–2021, severely decreased access to medical services deemed “non-essential,” including anti-VEGF agents. Utilization of these medications drastically fell during the beginning of the pandemic in many countries, including the United States [21–23]. However, some data suggests that most ophthalmic procedures, including anti-VEGF injections, returned to pre-pandemic averages by June of 2020 [24].

Despite these potential influences on national anti-VEGF implementation patterns, there have been few large-scale studies examining national anti-VEGF utilization in the last seven years. Given the potential shifts in anti-VEGF use due to the introduction of new medications, an ever-aging population, new clinical trials, pricing disparities, burgeoning biosimilars, and the COVID-19 pandemic, among other drivers of change, we sought to characterize national anti-VEGF treatment patterns. Our analysis aimed to update the body of existing literature with a new description of long-term anti-VEGF usage in light of the impacts of the aforementioned factors. We examined the diseases targeted for therapy, demographic data, and usage trends of bevacizumab, ranibizumab, aflibercept 2 mg, aflibercept 8 mg, brolucizumab, and faricimab from 2017 to 2023 in a large population of Medicaid, commercially insured, Medicare (including Part B and Medicare Advantage), and self-pay patients.

## Methods

### Data source

Data for this study were obtained from the Epic Cosmos [25], a nationally representative, HIPAA-compliant data set composed of de-identified data available in the electronic health record (EHR) from 1301 hospitals and over 28000 clinics across 50 states, representing over 238 million patients. Using this data set allowed us to obtain pooled encounter-specific information, such as patient demographics (age, gender, race etc.), orders placed, billing codes, visit diagnoses, and financial payor associated with a visit. To our knowledge, this is the first study using this data set to evaluate the trends of anti-VEGF injections given in ophthalmology clinics over the last seven years.

### Ethics statement

Compliant with United States Department of Health and Human Services (HHS) and New York University IRB/ethics board guidelines, IRB review was waived, as this study uses only completely de-identified information from a publicly available database as per HHS 45 CFR §46.102. There was no code or link to personal identifiers or protected health information and authors had no access to information that could identify subjects. Written and oral consent was also waived for the above reasons, as this research does not qualify as human subjects research as per HHS and New York University guidelines and it would not be feasible to obtain consent given the entirely anonymized nature of the dataset.

### Study sample

The Cosmos database was queried in January 2024 for encounters with an intravitreal injection current procedural terminology (CPT) code 67028 between January 1, 2017 and December 31, 2023. These encounters were then categorized by medications ordered in clinic, including faricimab-svoa, aflibercept 2 mg, aflibercept 8 mg, ranibizumab 0.3 mg, ranibizumab 0.5 mg, ranbiziumab-eqrn, ranibizumab-nuna, bevacizumab, and brolucizumab. Thus, if a patient received more than one injection in an encounter, each order would count as an injection. Each encounter associated with a medication order was then further categorized by visit diagnosis and primary financial payor. 4 categories of visit diagnoses of interest (retinal vascular occlusions, diabetic eye disease, exudative macular degeneration, or other) were used from established diagnoses groupers in Cosmos or by grouping ICD-10 code text descriptors (see Table 1). Financial payors included Medicaid, Medicare (including part B and Medicare Advantage), self-pay, or other payor (commercial/miscellaneous insurance). Some encounters were associated with multiple diagnoses or primary payors, but these redundancies accounted for less than 5% of all encounters and were included in our analyses. To fully understand the demographic characteristics of our study population, we also obtained information regarding age at the time of visit, legal sex, and self-identified race.

**Table 1:** ICD-10 codes of each diagnosis category.

Retinal Vascular Occlusions	Diabetic Eye Disease	Exudative Macular Degeneration
H34.8310, H34.8320, H34.8330, H34.8390, H34.8311, H34.8321, H34.8331, H34.8391, H34.8110, H34.8120, H34.8130, H34.8190, H34.8111, H34.8121, H34.8131, H34.8191, H34.9	E10.3211, E10.3212, E10.3213, E10.3219, E11.3211, E11.3212, E11.3213, E11.3219, E08.3211, E08.3212, E08.3213, E08.3219, E13.3211, E13.3212, E13.3213, E13.3219, E10.3311, E10.3312, E10.3313, E10.3319, E11.3311, E11.3312, E11.3313, E11.3319, E08.3311, E08.3312, E08.3313, E08.3319, E08.3411, E10.3411, E11.3411, E13.3411, E08.3412, E10.3412, E11.3412, E13.3412, E08.3413, E10.3413, E11.3413, E13.3413, E08.3419, E10.3419, E11.3419, E13.3419, E08.3511, E10.3511, E11.3511, E13.3511, E08.3512, E10.3512, E11.3512, E13.3512, E08.3513, E10.3513, E11.3513, E13.3513, E08.3519, E10.3519, E11.3519, E13.3519, E08.311, E10.311, E11.311, E13.311	H35.3211, H35.3221, H35.3231, H35.3291, H35.3213, H35.3223, H35.3233, H35.3293, H35.3211, H35.3220, H35.3230, H35.3290, H35.30

ICD-10 codes of visit diagnosis categories included in the study: retinal vascular occlusions, diabetic eye disease, and exudative macular degeneration.

### Statistical analysis

To calculate the rate of injections of a drug, we obtained the total number of ophthalmology clinic encounters per year. The gross number of injections per year both overall and per category (visit diagnoses vs financial payor) was divided by the total number of encounters in ophthalmology to find the injection rate per 100000 ophthalmology encounters (OE), Note that our study reported the rate of anti-VEGF use as a rate of injections per 100000 encounters due to the character of the data provided by Epic Cosmos. That is, total number of injections and ophthalmology encounters (across different diagnosis codes and with different linked anti-VEGF medications) is provided in this dataset (as opposed to injection rate per a set volume of patients). T-tests were used to quantify differences in change of rates of injections overall, across diagnoses, and across financial payors. All statistical analyses were carried out using IBM SPSS Statistics (Version 29).

## Results

**Fig 1:**
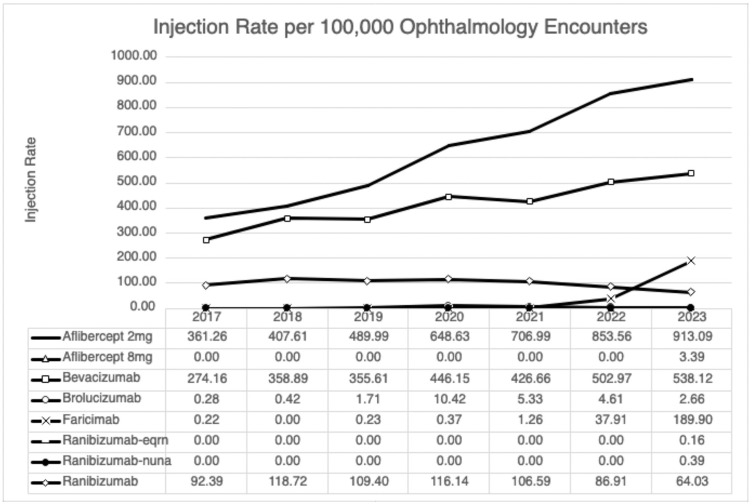
Injection rate per 100000 encounters. Injection rates per 100000 ophthalmology injection encounters of all medications from 2017-2023.

Table 2 includes demographic data of our study population. From 2017–2023, our study included 571545 cumulative injections as coded in Epic Cosmos across all encounters with 53.6% aflibercept 2 mg (306,247/571,545; 625.8/100000 OE), 34.8% bevacizumab 1.25 mg (198,696/571,545; 414.7/100000 OE), 7.83% ranibizumab 0.3 mg and 0.5 mg (44,803/571,545; 99.2/100000 OE), and 3.43% faricimab 6 mg (19,624/571,545; 32.8/100000 OE). Brolucizumab 6 mg, biosimilars ranibizumab-eqrn 0.5 mg and ranibizumab-nuna 0.5 mg, and aflibercept 8 mg all accounted for <1% of all total injections during this period (Table 3). From 2017 to 2023 the rate of aflibercept 2 mg increased from 361.3 injections/100000 OE in 2017 to 913.1 in 2023, the rate of bevacizumab increased from 274.2 to 538.1, and the rate of ranibizumab decreased from 92.4 to 64.0 (Fig 1). The rate of faricimab increased from 37.9 in 2022 to 189.9 injections/100000 OE while aflibercept 2 mg and bevacizumab increased from 853.6 to 913.1 and 503 to 538.1 in 2022 and 2023, respectively.

**Table 2:** Patient demographics.

Patient Demographics	
**Age**	**N (%)**
18-45 years	22372 (3.9%)
46-65 years	131870 (23.1%)
>65 years	417303 (73.0%)
**Legal Sex**	
Female	315395 (55.2%)
Male	256114 (44.8%)
Unknown/X	36 (<0.01%)
**Self-Identified Race**	
White	441373 (77.2%)
Black	57049 (9.98%)
Asian	12092 (2.12%)
Native American	4121 (0.72%)
Other or None	56910 (9.95%)
**Primary Financial Payors**	
Commercial/Miscellaneous	231570 (40.3%)
Medicare (Part B + Advantage)	309130 (53.7%)
Medicaid	19780 (3.44%)
Self-Pay	5722 (0.99%)
Other	9054 (1.57%)

Patient age, legal sex, self-identified race, and financial payors. Note: encounters may have more than one associated primary financial payor.

**Table 3:** Cumulative injections per year per anti-VEGF agent.

	Aflib 2 mg	Aflib 8 mg	Bevac	Brol	Fari	Ranib 0.3 mg	Ranib 0.5 mg	Ranib eqrn	Ranib nuna
Year									
2017	18299	0	13887	14	11	513	4167	0	0
2018	22547	0	19852	23	0	810	5757	0	0
2019	29775	0	21609	104	14	915	5733	0	0
2020	38339	0	26371	616	22	1113	5352	0	0
2021	50407	0	30420	380	90	1286	6314	0	0
2022	67620	0	39846	365	3003	1515	5370	0	0
2023	79260	294	46711	231	16484	1191	4367	14	34
Total	306247	294	198696	1733	19624	7343	37460	14	34

Number of total injections ordered across all patient encounters per year for aflibercept 2 mg (Aflib 2 mg), aflibercept 8 mg (Aflib 8 mg), bevacizumab (Bevac), brolucizumab (Brol), faricimab (Fari), ranibizumab 0.3 mg (Ranib 0.3 mg), ranibizumab 0.5 mg (Ranib 0.5 mg), ranibizumab-eqrn (Ranib eqrn), and ranibizumab-nuna (Ranib nuna).

As aflibercept 2 mg, bevacizumab, and ranibizumab comprised the large majority of all injections in the earlier part of our study period, we also performed overall analysis between all classes of anti-VEGF agents, with pooled injection rates (across all payors and indications) separated into 2 buckets: 2017–2021 (5 years) and from 2022 to the end of 2023 (2 full years). These analyses revealed that prior to 2022, aflibercept 2 mg had the highest rate (536.3 per 100000 encounters), followed by bevacizumab (377.4), ranibizumab 0.5 mg (93.3) and 0.3 mg (15.6), and lastly brolucizumab at a rate of 4.2 per 100000 OE. However, over the last 2 full years of our study, the increase in faricimab usage (after its approval in January 2022) becomes clear. From January 2022 to the end of December 2023, the rate of aflibercept 2 mg use jumped to 884.7 injections per 100000 OE, bevacizumab to 531.3, and the rate of faricimab was 117.4 in its first 2 years on the marketplace. Ranibizumab 0.5 mg had fallen to 58.65, while ranibizumab 0.3 mg was nearly unchanged at 16.3. Brolucizumab had decreased slightly to 3.6 over this period, while newly approved agents/doses aflibercept 8 mg (1.8/100000 OE) and ranibizumab-nuna (2.1) were scarcely utilized during 2022 and 2023.

NAMD was the most common diagnosis at 53.2% (311672/585056 total diagnoses across all encounters), followed by diabetic eye diseases at 26.9% (157187/585056), retinal vascular occlusion at 14.4% (84442/585056), and “other” at 5.43% (31755/585056). The most common “other” diagnoses included unspecified macular edema, unspecified retinal neovascularization, and cystoid macular edema. 53.7% of encounters listed Medicare as the primary payor (309130/575256 total primary payors), while commercial/miscellaneous insurance covered 40.2% (231570/575256), and Medicaid covered 3.44% (19780/575256).

In patients with nAMD, aflibercept 2 mg was used at a mean rate of 353.5 per 100000 OE (SD = 101.8) followed by bevacizumab at 202.8 (SD = 32.3) [t(6)=5.35, p = 0.002] and ranibizumab 0.5 mg at 62.9 per 100000 OE (SD = 16.6) [t(6) = 6.62, p < 0.001] from 2017–2023. For DR patients, rates of administrations of bevacizumab were 124.6 per 100000 OE (SD = 42.1) and aflibercept 2 mg was 168.7 (SD = 61.9) [t(6)=5.03, p = 0.002]. The overall use of these two medications exceeded all others from 2017–2023. The third most common drug for DR was ranibizumab 0.3 mg, used at a rate of 14.2 per 100000 OE. For patients with RVO, use of aflibercept 2 mg was 96.0 per 100000 OE (SD = 47.8) and that of bevacizumab was 61.1 per 100000 OE [t(6)=2.51, p = 0.046] from 2017–2023. In these patients, ranibizumab 0.5 mg was the third most common given medication at a rate of 14.0 per 100000 ophthalmology encounters.

The primary financial payor associated with each ophthalmology encounter was also analyzed. Patients with Medicare (including both Medicare Advantage and part B [traditional fee-for-service Medicare]) insurance most often received aflibercept 2 mg at significantly higher rates than bevacizumab (M = 364.3/100000 OE, SD = 109.7 vs M = 187.1/100000 OE, SD = 28.4, respectively) [t(6) = 5.67, p = 0.001]. Medicare insurance patients’ rates of ranibizumab 0.5 mg (M = 62.2/100000 OE) were greater than that of ranibizumab 0.3 mg (M = 6.73/100000 OE) and faricimab (19.4/100000 OE from 2017–2023. For patients with commercial insurance, there was no significant difference in the average rate of use of aflibercept 2 mg (M = 233.9/100000 OE, SD = 94.9) and bevacizumab (M = 197.9/100000 OE, SD = 56.2) [t(6)=2.07, p > 0.05]. Ranibizumab 0.5 mg was also a commonly administered medication (19.9/100000 OE), and its use was greater than that of faricimab (12.5/100000 OE) and ranibizumab 0.3 mg (6.27/100000 OE). Medicaid patients had a marginally significant difference in use of bevacizumab (M = 19.9/100000 OE, SD = 7.32) and aflibercept 2 mg (16.1/100000 OE, SD = 7.22) [t(6)]=2.95, p = 0.026], and these were far greater used than other drugs (ranibizumab 0.3 mg = 1.97/100000 OE, ranibizumab 0.5 mg = 0.84/100000 OE, and faricimab (0.47/100000 OE). Among self-pay patients, aflibercept 2 mg was more commonly used (M = 7.15/100000 OE, SD = 2.26) compared to bevacizumab (M = 3.44/100000 OE, SD = 2.48) [t(6)=7.39, p < 0.001], while the use of ranibizumab 0.5 mg, ranibizumab 0.3 mg, and faricimab were negligible.

## Discussion

From January 1, 2017 to December 31, 2023, the absolute number of aflibercept, bevacizumab, and faricimab (since its approval in 2022) injections increased every year, whereas brolucizumab, and ranibizumab 0.3 mg and 0.5 mg had more variable courses. Aflibercept 2 mg and bevacizumab injection rates increased from 2017–2023, while the rate of ranibizumab increased initially but then decreased by approximately one third from 2017–2023. While ranibizumab use lagged, faricimab had a greater than 5-fold utilization rate increase from 2022 to 2023 and surpassed ranibizumab use in 2023.

From 2017–2023, the utilization rate of aflibercept 2 mg (the most used agent overall) remained higher than bevacizumab (the second most frequently used agent) which corresponds to trends from prior recent studies [10], and is a change from 2015 when bevacizumab was the most used anti-VEGF drug [5]. However, from 2019 onwards, the rate of use of aflibercept 2 mg increased much more than bevacizumab. Although there is still debate of the superiority of each drug (each of which has high efficacy), the recent preference for aflibercept 2 mg may be due to recent data from large clinical trials. DRCR Network Protocol T, which was published in 2015, demonstrated superior visual outcomes from aflibercept when compared to bevacizumab among eyes with DME and a baseline acuity worse than 20/40 [7]. Protocol AB (published in 2020) showed the non-inferiority of aflibercept treatment compared to panretinal photocoagulation and vitrectomy in the treatment of vitreous hemorrhage in PDR, and Protocol W (published in 2021) found that aflibercept significantly reduced the development of center involving (CI) DME and PDR in patients with severe non-proliferative DR [26,27]. Additionally, despite similar visual outcomes in central retinal vein occlusions, the frequency and total number injections was significantly reduced in aflibercept patients and there was significant improvement in retinal nonperfusion in aflibercept eyes compared to bevacizumab [28]. Clearly, clinical trial data supports the use of aflibercept in DME in patients with poor vision, and the choice of the DRCR network using aflibercept as the gold standard of anti-VEGF treatment may have signaled its place as the preferred drug. As the treatment of DR has shifted toward anti-VEGF use (instead of lasers or surgery) following publication of DRCR protocol S, we would expect a continued rise of its use [9]. It is important to note, however, that the SCORE2 trial showed no difference in visual acuity outcomes for retinal vein occlusions between aflibercept 2 mg and bevacizumab (although there was better resolution of subretinal and intraretinal fluid on average), and similarly, DRCR Protocol AC demonstrated that patients with DME and vision worse than 20/40 had similar outcomes whether initially treated with aflibercept 2 mg or with bevacizumab and then switched to aflibercept 2 mg if there was an inadequate response [29,30].

Our study is the first to analyze patterns in the adoption of newer agents introduced in the past 5 years, including faricimab, brolucizumab, ranibizumab-nuna, ranibizumab-eqrn, brolucizumab, and aflibercept 8 mg. While our results demonstrate that aflibercept 2 mg and bevacizumab remain the most utilized agents overall across all indications, there is a strong demand for the development of agents with greater or equivalent therapeutic efficacy with less treatment and/or financial burden.

We observed a rapid adoption of faricimab, which surpassed the utilization of ranibizumab after less than 2 years following FDA approval in January 2022. Intravitreal faricimab holds the promise of increased efficacy with less treatment burden for patients, as it has dual action on both angiopoetin-2 and VEGF [3]. The TENAYA and LUCERENE phase III trials demonstrated that the majority of patients receiving faricimab fared well on 12 week or longer intervals based on macular central subfield thickness and have non-inferior visual outcomes compared to aflibercept 2 mg at 8-week intervals for nAMD [31]. Similarly, in the YOSEMITE and RHINE phase III studies, the faricimab arm demonstrated non-inferior visual acuity at longer treatment intervals compared to aflibercept 2 mg every 8 weeks for DME [32]. Because it may alleviate a major burden and treatment hurdle for patients on treatment regimens with anti-VEGF agents as it can avoid the need for recurrent, frequent injections, it is not surprising that faricimab use has sharply risen in tandem with the decline of ranibizumab in our study.

Unlike faricimab, biosimilars (currently of ranibizumab) comprised less than 1% of anti-VEGF utilization. Previous research has demonstrated that biosimilars may increase costs to the health care system and patients [33], although ranibizumab biosimilars have shown similar clinical outcomes to a much lower cost bevacizumab [34,35]. Once aflibercept biosimilars come to the market, it is likely that the utilization of biosimilars may change significantly, given the apparent preference for aflibercept 2 mg use based upon efficacy from trial data, clinical experience/opinions on efficacy, and appropriate pricing (either lower cost or with a favorable reimbursement structure).

Brolucizumab has not experienced widespread adoption like faricimab due to reported safety concerns, with the most severe being visual loss secondary to retinal vasculitis. This drug was anticipated as a longer activating agent or an alternative for patients with non-responsive nAMD as it has a less frequent dosing schedule, similar to faricimab. The promising results from the brolucizumab clinical registration trials such as HAWK and HARRIER, as well as the early real-world study (BREW), demonstrated significant improvement in mean central subfoveal thickness in patients who were previously treated with anti-VEGFs [36,37]. The safety of this medication came into question, however, when there were numerous reports of intraocular inflammation in practice. This was confirmed during the MERLIN trial, in which retinal vasculitis and retinal vascular occlusion were reported with a higher frequency in the brolucizumab arms, leading to early study termination [4,38].

Aflibercept 8 mg was approved late in our study and does not have a permanent J code as of March 1, 2024 for billing, which limits physicians from utilizing a high-cost medication for concerns of denied reimbursement. Studies will likely be needed after the adoption of a permanent J code to properly ascertain the utilization of aflibercept 8 mg.

The most common primary financial payors in our data were Medicare and commercial/miscellaneous insurance. While bevacizumab and aflibercept 2 mg were the most used drugs in patients with these insurance types, Medicare patients had a large difference in the usage of aflibercept 2 mg and bevacizumab while commercial insurance did not. In the past, bevacizumab has been shown to be the primary drug used for exudative AMD in Medicare patients [39,40], especially after the release of the Comparison of Age-related macular degeneration Treatment Trial (CATT) in 2011 [41]. Because bevacizumab was shown to be equally effective as ranibizumab in visual outcomes for AMD and was associated with a much lower treatment cost (and higher savings for patients with Medicare), it is not surprising that it was highly used [42]. However, we found that bevacizumab was used less in patients with Medicare and equally to aflibercept 2 mg in commercially insured patients in the last seven years. The choice of anti-VEGF medication for patients is a complex decision that is influenced by many factors unique to each patient. An article interviewing retinal specialists described the dilemma physicians face when deciding which treatment to offer patients with AMD, as they must consider insurance coverage in addition to clinical trial data. Some commercial insurance plans have a required “step-edit” that compels providers to initiate treatment with bevacizumab or risk removal from a plan [43]. This may have contributed to our observation of similar usage of aflibercept and bevacizumab in patients with primary commercial insurance coverage.

The increased use of aflibercept 2 mg when compared to bevacizumab in Medicare patients could be explained by several factors. A recent study by Dickson and James in AMD patients with Medicare part B found that ophthalmologists who accepted manufacturer payments were significantly more likely to prescribe higher cost therapies [44], similar to a prior study in 2017 of Centers for Medicare and Medicaid Services (CMS) data [45]. Other articles suggest the overall higher availability of aflibercept, limited access to compounding pharmacies that produce bevacizumab, and the variable quality of the compounded agent could be responsible for the decline in bevacizumab usage [46]. Furthermore, a recent article investigating anti-VEGF trends in Medicare Part B patients (using data provided by CMS and therefore including over 2.5 million injections) reported a similar trend with aflibercept 2 mg utilization from 2012–2015, in the period just prior to our study [47]. This study found nearly a 70% increase in the use of aflibercept 2 mg among Medicare Part B patients across all indications in from 2012–2015, and a 17% decrease in bevacizumab usage (with bevacizumab still edging out aflibercept as the most used mediation nationally during the study period). Thus, the primary use of Medicare plans amongst our patients may have also contributed to the increased use of aflibercept 2 mg overall we observed, even though it has been shown to be less cost-effective than bevacizumab for patients with diabetic macular edema [48] or with exudative macular degeneration [49]. Further studies evaluating the usage of bevacizumab and higher cost agents such as faricimab and aflibercept 2 mg or 8 mg in patients with Medicare Advantage (Part C) vs. traditional fee for service Medicare (Part B) would be helpful to explain the difference in their usage. Although appropriate medication choice involves many factors and may vary with each patient, it is important for equity purposes that those with Medicare Advantage are not facing inequitable restrictions on access to therapeutics with step therapy. This is a clinical issue, an ethical issue, and a legal/equity issue, as Medicare Advantage “must provide the same benefits offered by Original Medicare, but may apply different rules, costs, and restrictions” [50].

Our study describes the trend in use of anti-VEGF medications from 2017–2023 using the unique Epic Cosmos database, which has not been used for research within ophthalmology until now. Although we used a robust database with patients from all 50 states and from large and small clinics, we were limited in that our study only included data from practices using Epic that send their data to Cosmos. As Cosmos is a de-identified database, we also could not obtain patient level data to correlate injection type with visit diagnosis and insurance payor at an individual level. Notably, this means that the stratification of diagnoses across each payor class (i.e., percent of nAMD among commercial payors versus Medicare, etc.) was not possible given the constraints of the data collected by Epic Cosmos. Furthermore, although Epic holds the largest market share in the electronic health record market in America, it is not known if there are geographic or socioeconomic biases to practices that utilize Epic and/or those that choose to opt-in to Cosmos. The proportion of retina physicians in America utilizing Epic systems is also unknown. Solo practitioners or smaller practices, which may be captured in other datasets such as the American Academy of Ophthalmology Intelligent Research in Sight (IRIS) registry, may be less likely to be included in Cosmos given the diversity of outpatient electronic medical record systems. Despite these limitations, this nationally representative and extensive data source allowed us to explore trends in anti-VEGF use across several types of insurance payors and with newer drugs as recently as 2023. We found a momentous paradigm shift: aflibercept 2 mg now accounts for the majority of anti-VEGF use in the Epic Cosmos database, overall utilization of aflibercept, bevacizumab, and faricimab is growing, and faricimab use has undergone the largest relative and absolute increase in rate of use from 2022 to 2023.

## Abbreviations

anti-VEGF: Anti-vascular endothelial growth factor
OE: ophthalmology encounters
nAMD: neovascular age related macular degeneration
DR: diabetic retinopathy
DME: diabetic macular edema
PDR: proliferative diabetic retinopathy
RVO: retinal vein occlusion
FDA: Food and Drug Administration
PRP: panretinal photocoagulation
WAC: wholesale acquisition cost
CPT: current procedural terminology.
